# Protective Effect of Nalbuphine Combined With Dexmedetomidine on the Lungs of Children With Atelectasis and Foreign Body in the Bronchus During the Perioperative Period

**DOI:** 10.3389/fphys.2022.799183

**Published:** 2022-05-05

**Authors:** Yuan Wang, Hong-Yang Han, Ying-Ping Jia, Li-Yuan Zhao, Ying Li, Bian-Jing Zuo, Jie Zhang

**Affiliations:** ^1^ Department of Anesthesiology, Children’s Hospital Affiliated with Zhengzhou University, Zhengzhou, China; ^2^ Department of Radiology, Zhengzhou Central Hospital, Zhengzhou, China; ^3^ Department of Otolaryngological, Children’s Hospital Affiliated with Zhengzhou University, Zhengzhou, China; ^4^ Department of Ultrasound, Children’s Hospital Affiliated with Zhengzhou University, Zhengzhou, China; ^5^ Department of Anesthesiology, Zhengzhou University First Affiliated Hospital, Zhengzhou, China

**Keywords:** nalbuphine, dexmedetomidine, atelectasis, foreign body in the bronchus, child, lung protection

## Abstract

**Objective:** The present study aims to explore the protective effect of nalbuphine combined with dexmedetomidine on the lungs of children with atelectasis who have a foreign body in a bronchus during the perioperative period.

**Methods:** A total of 180 patients whose computed tomography scan showed atelectasis and a foreign body in a bronchus were randomly divided into three groups: group C (conventional anesthesia group), group D (dexmedetomidine group), and group N + D (nalbuphine combined with the dexmedetomidine group). The following indicators were recorded: 1) heart rate (HR) and mean arterial pressure (MAP) prior to induction (T_0_), at bronchoscope placement (T_1_), at intubation after surgery (T_2_), at tube removal (T_3_), 10 min after tube removal (T_4_), 20 min after tube removal (T_5_), and at awaking (T_6_); 2) monocyte toll-like receptors (TLRs) TLR⁃2, TLR⁃4, tumor necrosis factor α, interleukin 6, oxygenation index, and the B-line sum at T_0_, T_3_, 2 h (T_7_), and 24 h (T_8_) after tube removal; and 3) hospital stay after surgery.

**Results:** Compared with group C, in group D and group N + D, 1) the HR and MAP at T_1_∼T_6_ were lower; 2) the inflammatory factor indicator and B-line sum were lower, and the oxygenation index was higher at T_7_ and T_8_; 3) the agitation and cough scores were decreased during tube removal; and 4) the Ramsay sedation score was higher, and ventilator weaning time was shortened at T_4_∼T_6_ (*p* < 0.05). Compared with group D, in group N + D, 1) the inflammatory factor indicator and B-line sum were lower at T_8_; 2) the oxygenation index was higher (*p* < 0.05). Compared with groups C and D, in group N + D, the length of hospital stay was decreased (*p* < 0.05).

**Conclusion:** In patients with atelectasis and a foreign body in a bronchus during the perioperative period, nalbuphine combined with dexmedetomidine may be capable of reducing the oxidative stress response, improving the oxygenation index, decreasing the pulmonary fluid content, protecting the lung, and facilitating postoperative recovery.

## Introduction

The presence of a foreign body in a bronchus is a common pediatric emergency, and mechanical obstruction by a large foreign body may result in atelectasis. In the process of removing the foreign body, reoxygenation after hypoxia caused by pulmonary re-expansion and stress stimulation in the perioperative period may aggravate the lung injury and affect the prognosis. According to relevant domestic and foreign reports, dexmedetomidine can inhibit sympathetic nerve activity and catecholamine release, reduce the synthesis and release of pro-inflammatory factors [e.g., tumor necrosis factor α (TNF-α) and interleukin 6 (IL-6)], inhibit the inflammatory response, improve lung function, and protect the lung (Giovannitti et al., 2015; Zhang, 2018; Quan, 2019). However, the inhibition of reoxygenation after hypoxia during lung re-expansion has not been reported.

Nalbuphine mainly activates κ opioid receptors and produces spinal analgesia (He et al., 2021). Because of its long duration and few adverse reactions, it can be used before operations (Xu et al., 2019) to effectively prevent anesthesia stimulation and central sensitization caused by operation trauma as well as reduce the intraoperative inflammatory response (Li et al., 2016).

Based on the preliminary results of the previous study, (Wang and Jia, 2015), the protective effect of nalbuphine preemptive analgesia combined with intraoperative dexmedetomidine on lung re-expansion in children with atelectasis after foreign body removal was explored. The exploration was conducted through the expression of monocyte toll-like receptors (TLRs), TLR2 and TLR4, in peripheral blood during the perioperative period, the influence of inflammatory factors (e.g., TNF-α and IL-6) and the oxygenation index, and the extravascular pulmonary fluid content assessment with a B-line sum (Picano and Pellikka, 2016).

## Materials and Methods

The present prospective, randomized, controlled, and double-blind clinical study was approved by the Medical Ethics Committee of the Children’s Hospital Affiliated with Zhengzhou University (2021-K-98). The family members of the children all signed the informed consent form.

## Subject

From March 2, 2020, to March 31, 2021, a total of 180 children with atelectasis and a foreign body in a bronchus diagnosed *via* a computed tomography (CT) scan were enrolled in the present study. The patients underwent removal of the foreign body under rigid bronchoscopy; the patients were instructed to fast for 6 h before the operation and forbidden to drink water 2 h before the operation.

Inclusion criteria: children with CT-confirmed atelectasis and a history of foreign body inhalation in the last 2 weeks (foreign bodies: plant seeds such as peanuts or melon seeds).

Exclusion criteria: 1) children who experienced a failure to remove the foreign body during the operation or children in whom a postoperative CT confirmed a failure to completely remove the foreign body; and 2) children with pulmonary consolidation, congenital heart disease, heart failure, respiratory failure, or cyanosis.

The patients were randomly divided into three groups according to the random number table: group C (the conventional anesthesia group), group D (the dexmedetomidine group), and group N + D (the nalbuphine combined with dexmedetomidine group) (*n* = 60, each).

## Anesthesia Method

**FIGURE 1:**
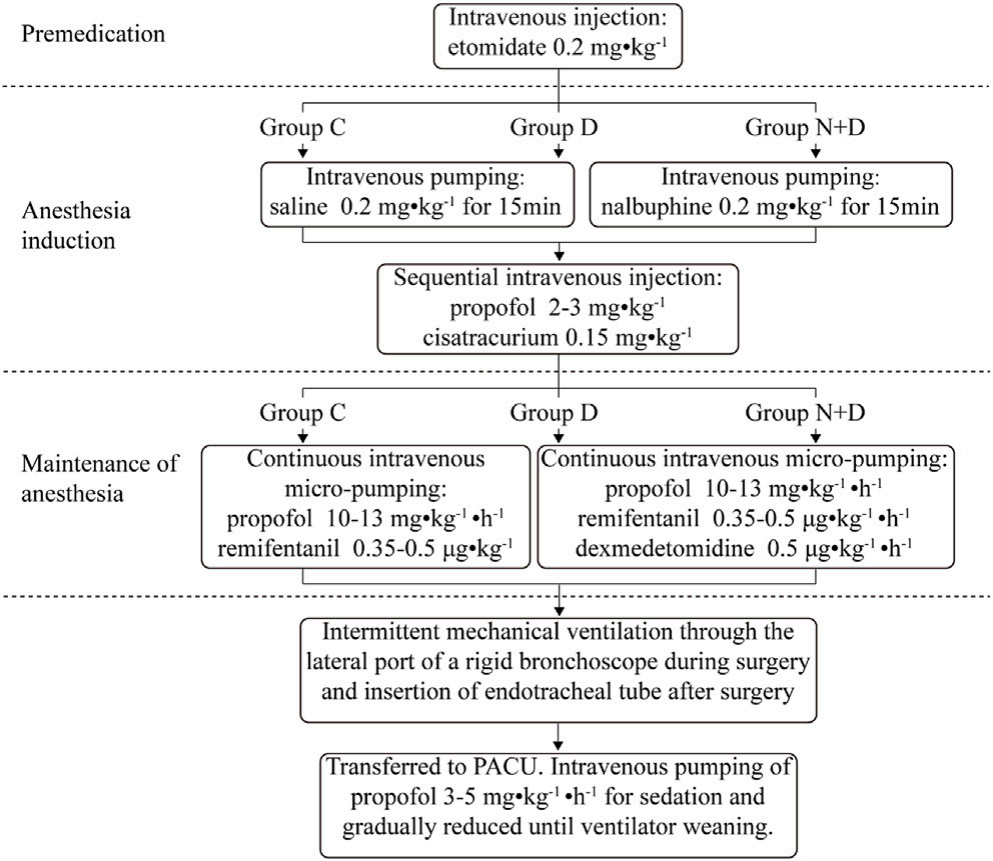
Detailed anesthesia method for the removal of the foreign body under rigid bronchoscopy. All patients sequentially underwent the procedures of premedication, anesthesia induction, maintenance of anesthesia, and anesthesia recovery. During the premedication procedure, nalbuphine was administered in group N + D; an equal amount of normal saline was administered in group D and group C. During anesthesia maintenance, group N + D and group D received dexmedetomidine until the end of the operation and group C received an equal amount of normal saline for maintenance.

All patients sequentially underwent the procedures of premedication, anesthesia induction, anesthesia maintenance, and anesthesia recovery. During the premedication procedure, group N + D received nalbuphine and group D and group C received an equal amount of normal saline. During anesthesia maintenance, group N + D and group D were administered with dexmedetomidine until the end of the operation, and group C was administered with an equal amount of normal saline for maintenance. The detailed procedures were as follows (Figure 1).

### Premedication

All the patients were slowly intravenously administered with etomidate 0.2 mg kg^−1^ and conveyed to the operating theatre after falling asleep.

### Anesthesia Induction

Nalbuphine 0.2 mg kg^−1^ was diluted to 20 ml with normal saline, and intravenous pumping was completed within 15 min. Intravenous injection of propofol 2–3 mg kg^−1^, cisatracurium 0.15 mg kg^−1^, and remifentanil 2–3 μg kg^−1^ was performed for induction. After the value of Narcotrend deceased to D_2_, a rigid bronchoscope was placed, and a Primus anesthesia machine (Druger, Germany) was connected for intermittent mechanical ventilation (controlling breathing during the microscopic examination and stopping breathing control during foreign body removal) in the pressure control mode through the side hole of the bronchoscope [FiO_2_ 40–60%, flow 3 L min^−1^, RR 30–40 times•min^−1^, V_T_ 8–10 ml kg^−1^, SpO_2_ ≥ 95%, and PetCO_2_ 35–45 mmHg (1 mmHg = 0.133 kPa)].

### Anesthesia Maintenance

The anesthesiologists in the present study were blind to the grouping. After anesthesia induction, 10 ml kg^−1^ of compound electrolyte solution containing 1% glucose was injected intravenously into all children. Immediately, continuous intravenous micro-pumping of propofol 10–13 mg kg^−1^•h^−1^ was performed during each operation in order to maintain the Narcotrend value at D_2_–E_1_ and remifentanil value at 0.35–0.5 μg kg^−1^•min^−1^ until the end of the operation. Intravenous pumping of dexmedetomidine 0.5 μg kg^−1^•h^−1^ was conducted until the end of the operation. After the operation, a tracheal tube was inserted, and the patient was transferred to the recovery room.

### Anesthesia Recovery

The Siemens (Maquet SERVO-i) ventilator was connected in the SIMV + PSV mode to control ventilation: a flow of 3 L min^−1^, FiO_2_ of 60–40%, initial RR of 20–25 times•min^−1^, and V_T_ of 8–10 ml kg^−1^. Intravenous pumping of propofol 3–5 mg kg^−1^ was conducted for sedation and gradually reduced until ventilator weaning. The ventilator is adjusted until the following conditions are met: 1) suction support pressure < 10 cm H_2_O; 2) positive end-expiratory pressure (PEEP) < 5 cm H_2_O; and 3) FiO_2_ < 50% (Sun et al., 2018).

The ventilator weaning test was conducted according to the spontaneous breathing trial (SBT) standard. In the case of a successful trial, a simple breathing bag was used for ventilation; in the case of an unsuccessful trial, the current ventilation mode was continued for 10 min, and then, SBT was conducted again until ventilator weaning.

After ventilator weaning, propofol pumping was stopped. After the swallowing reflex was recovered, spontaneous respiration was stable, air absorption could be maintained at SpO_2_ ≥ 95%, the respiratory tract was fully cleared, and the tube was removed.

### Indicator Monitoring and Recording

1) Respiratory circulation indicators: heart rate (HR), mean arterial pressure (MAP), PetCO_2_, and SpO_2_ were monitored during each operation. The HR and MAP were recorded at the following time points: prior to induction (T_0_), at bronchoscope placement (T_1_), at intubation after surgery (T_2_), at tube removal (T_3_), 10 min after tube removal (T_4_), 20 min after tube removal (T_5_), and at awaking (T_6_).

An HR and MAP of <30% of the sober-state HR and MAP were defined as bradycardia and hypotension. Atropine 0.01 mg kg^−1^ and ephedrine 0.3 mg kg^−1^, respectively, were injected intravenously.

An HR and MAP of >30% of the sober-state HR and MAP were defined as tachycardia and hypertension. Remifentanil 0.5–1 μg kg^−1^ was injected intravenously, and the factors were recorded as adverse events in the circulatory system.

An SpO_2_ of <90% was defined as hypoxemia. In such cases, it was necessary to increase the fraction of inspired oxygen and manually control positive pressure ventilation (*p* ≤ 30 cm H_2_O).

A PetCO_2_ of >60 mmHg was defined as hypercapnia. In such cases, it was necessary to adjust respiration parameters and record the PetCO_2_ as an adverse event in the respiratory system.

2) Collection and detection of inflammatory factors: a volume of 3 ml peripheral venous blood was collected in T_0_, T_3_, T_7_, and T_8_, respectively. The supernatant was taken after the blood was centrifuged for 20 min at 3,000 r/min, gathered in the EP tube, and kept in a −20°C refrigerator for centralized measurement in order to avoid repeated freezing and thawing.

The TNF-α, IL-6 (kit provided by eBioscience), TLR⁃2, and TLR⁃4 (kit manufactured by RayBiotech) were detected with double antibody sandwich ELISA by using the MICRO LAB FAME automatic enzyme immunity analyzer of HAMILTON. The optical density of the sample was obtained by colorimetric analysis, and the optical density values of the test sample were converted to the concentration values (TLR⁃2 and TLR⁃4: ng/ml; TNF⁃α and IL-6 pg/ml) according to the standard curve. The operation process was carried out in strict accordance with the reagent specifications.

3) Oxygenation index: blood samples from radial arteries were taken at T_0_, T_3_, T_7_, and T_8_ for blood gas analysis, and the oxygenation index (PaO_2_/FiO_2_) was calculated.

4) Assessment of pulmonary fluid content by ultrasound B-line sum: the Sonosit M-Turbo portable color ultrasound machine was used to divide the lung into anterior, lateral, and posterior areas by using anterior and posterior axillary lines as the boundaries. Next, each lung was divided into upper and lower lung fields using the connection line between the two nipples, dividing the bilateral lung into 12 areas (Liu et al., 2019).

The B-line was measured using a high-frequency line array probe (6.5–7.5 MHZ) through the bilateral chest wall in the supine position at T_0_, T_3_, T_7_, and T_8_ (Picano and Pellikka, 2016). The B-line sum was counted.

5) Agitation scores: the agitation score at T_3_ was recorded (0 points: quietness and cooperativeness; 1 point: mild agitation, body agitation during aspiration of sputum, and intermittent moaning; 2 points: agitation without stimulus, continuous moaning, and a necessity to fix the upper limbs; and 3 points: violent struggling and shouting and the necessity to press limbs by an external force). An agitation score of 0–1 points was considered satisfactory.

6) Sedation scores: the Ramsay sedation score at 10 min (T_4_) and 20 min (T_5_) after tube removal and at awakening (T_6_) was recorded (1 point: anxiety and agitation; 2 points: quietness and cooperativeness; 3 points: response only to command, light touch, and normal sound; 4 points: quick response after gently patting on the eyebrows or upon loud stimulus in the ears; 5 points: slow response after gently patting on the eyebrows or upon loud stimulus in the ears; and 6 points: no response after gently patting on the eyebrows or upon loud stimulus in the ears). A score of 1 point indicated agitation; 2–4 points indicated that sedation was satisfactory; and 5–6 points indicated over-sedation. In patients with a score of ≥2 points at T_3_ or <2 points after tube removal, propofol 0.5∼1 mg kg^−1^ was given intravenously, and the cases of drug intervention were recorded.

7) Cough scores: the cough score at T_3_ was recorded (Pei et al., 2018) [grade 1: no cough; grade 2: mild cough (1–2 times) and smooth and steady tube removal; grade 3: moderate cough (3–4 times); grade 4: severe cough (5–10 times); and grade 5: agitation and inability to remove the tube].

8) The time from operation completion to ventilator weaning, the time from ventilator weaning to tube removal, and the time from ventilator weaning to awakening (Ramsay sedation score: 4 points) were recorded. The incidence of hypoxemia and airway spasms during the resuscitation period was analyzed, and the postoperative hospital stay was recorded.

## Statistical Method

The SPSS 25.0 software was used for data analysis. The measurement data conformed to a normal distribution and were expressed as mean ± standard deviation ( $\bar{x}$ ± s). One-way ANOVA was used for comparisons between the three groups, while the LSD test was used for two-way comparisons between groups. The χ^2^ test or Fisher’s exact test was used for enumeration data analysis. A *p* value of <0.05 was considered statistically significant.

## Results

### Demographics

A total of 180 patients were included in the present study; each patient group comprised 60 patients. In two children, there was a failure to remove the foreign body during the operation; hence, these patients were excluded from the study. There were no statistically significant differences among the groups (*p* > 0.05; Table 1).

**TABLE 1:** Demographics of patients in each group ( $\bar{x}$ *± s*).

	Group C	Group D	Group N + D	*p* value
Gender (male/female)	37/22	35/24	38/22	0.931
Age (months)	26 ± 16	25 ± 15	26 ± 15	0.772
Weight (kg)	14.9 ± 6.1	15.3 ± 5.6	14.7 ± 5.8	0.619
Time from onset to admission (days)	8.6 ± 4.2	8.3 ± 4.0	8.4 ± 3.9	0.796
Time from admission to operation (hours)	20.9 ± 11.6	20.2 ± 12.1	20.6 ± 11.9	0.823
Operation time (min)	21.2 ± 5.5	20.7 ± 6.3	22.1 ± 5.9	0.265

## Cycle Indicators and Adverse Events

Compared with group C, the HR and MAP decreased at T_1_–T_2_ (*p* < 0.05) and greatly decreased at T_3_–T_6_ (*p* < 0.01) in group D and group N + D. There were no statistically significant differences in HR and MAP between group N + D and group D at any of the time points (Table 2). No cases of hypotension and bradycardia in group C required intervention. There were two cases of bradycardia in group D and group N + D, respectively; however, there was no statistical significance (*p* > 0.05).

**TABLE 2:** Cycle indicators at different time points in each group ( $\bar{x}$ *±s*).

Group	Item	T_0_	T_1_	T_2_	T_3_	T_4_	T_5_	T_6_
C	HR (bpm)	116.6 ± 17.3	115.3 ± 16.9	109.2 ± 16.2	129.3 ± 14.9	125.3 ± 14.7	119.3 ± 14.4	127.3 ± 16.8
D		117.3 ± 16.5	94.7 ± 15.4^a^	94.3 ± 14.8^a^	93.2 ± 14.1^aa^	96.3 ± 13.6^aa^	94.3 ± 13.5^aa^	98.3 ± 13.7^aa^
N + D		116.5 ± 14.9	93.4 ± 15.1^a^	93.2 ± 14.7^a^	94.7 ± 14.1^aa^	95.4 ± 13.5^aa^	94.6 ± 13.6^aa^	97.3 ± 13.9^aa^
C	MAP (mmHg)	71.3 ± 4.3	77.7 ± 4.9	73.7 ± 4.7	87.5 ± 4.6	85.5 ± 4.4	84.5 ± 5.3	87.8 ± 4.6
D		70.9 ± 4.7	66.5 ± 5.4^a^	59.8 ± 5.3^a^	57.7 ± 4.6^aa^	56.6 ± 4.4^aa^	55.6 ± 4.6^aa^	57.3 ± 4.4^aa^
N + D		70.5 ± 4.0	65.7 ± 5.3^a^	59.0 ± 5.0^a^	56.7 ± 4.5^aa^	55.7 ± 4.9^aa^	55.0 ± 4.5^aa^	56.8 ± 4.0^aa^

a p < 0.05 compared with group C.

b p < 0.01 compared with group C.

There were five cases of hypoxemia in group C during the recovery period; among these, three were relieved after sputum aspiration, and the other two had transient airway spasms and were relieved by intensive sedation and PEEP. The remaining two groups had no adverse events in the respiratory system.

### Inflammatory Factor, Oxygenation Index, and B-Line Sum

Compared with group C, in group D and group N + D, the concentrations of TLR⁃2, TLR⁃4, TNF⁃α, and IL-6, as well as the B-line sum, were low at T_7_ and T_8_, and the oxygenation index was high (*p* < 0.05). Compared with group D, in group N + D, the B-line sum and inflammatory factor indicators at T_8_ were low, and the oxygenation index was high (*p* < 0.05; Table 3).

**TABLE 3:** Inflammatory factor, oxygenation index, and B-line sum indicator at different time points in each group ( $\bar{x}$ *±s*).

Group	Item	T_0_	T_3_	T_7_	T_8_
C	TLR-2 (ng/ml)	0.239 ± 0.128	0.288 ± 0.186	2.193 ± 0.212	2.192 ± 0.138
D		0.232 ± 0.133	0.244 ± 0.181	1.024 ± 0.312^a^	0.987 ± 0.156^a^
N + D		0.213 ± 0.142	0.231 ± 0.197	0.945 ± 0.326 ^a^	0.258 ± 0.136 ^aa^ ^,^ ^b^
C	TLR-4 (ng/mL)	1.088 ± 0.113	1.155 ± 0.275	3.016 ± 0.339	3.005 ± 0.274
D		1.092 ± 0.133	1.160 ± 0.264	2.002 ± 0.327^a^	1.824 ± 0.256^a^
N + D		1.101 ± 0.126	1.183 ± 0.250	1.960 ± 0.369^a^	1.220 ± 0.283^aa^ ^,^ ^b^
C	TNF⁃α (pg/ml)	15.3 ± 4.3	16.3 ± 3.6	32.6 ± 4.5	36.3 ± 4.7
D		15.4 ± 3.9	16.6 ± 3.4	24.4 ± 4.6 ^a^	30.3 ± 5.8^a^
N + D		15.6 ± 3.7	16.3 ± 3.8	23.3 ± 4.4 ^a^	16.6 ± 4.4^aa^ ^,^ ^b^
C	IL-6 (pg/ml)	13.3 ± 3.3	14.6 ± 4.2	31.5 ± 8.7	33.3 ± 9.7
D		13.5 ± 3.9	14.8 ± 3.4	25.6 ± 6.4^a^	23.3 ± 7.8^a^
N + D		12.9 ± 4.2	13.9 ± 3.6	23.9 ± 7.3^a^	14.2 ± 8.2^aa^ ^,^ ^b^
C	Oxygenation index	395 ± 38	397 ± 29	399 ± 32	408 ± 29
D		402 ± 27	403 ± 33	429 ± 21^a^	433 ± 22^a^
N + D		401 ± 25	405 ± 30	429 ± 22^a^	463 ± 23^aa^ ^,^ ^b^
C	B-line sum	2.0 ± 0.9	2.1 ± 1.0	3.9 ± 2.1	4.0 ± 2.0
D		2.1 ± 0.8	2.1 ± 0.9	3.1 ± 2.2^a^	3.0 ± 1.9^a^
N + D		2.0 ± 0.8	2.0 ± 0.9	2.9 ± 2.1^a^	2.3 ± 1.7^aa^ ^,^ ^b^

a p < 0.05 compared with group C.

^aa^p < 0.01 compared with group C.

^b^p < 0.05 compared with group D.

### Agitation Score at Tube Removal and the Ramsay Sedation Score

Compared with group C, in group D and group N + D, the agitation score decreased significantly at T_3_ (*p* < 0.01), and the score was high at T_4_, T_5_, and T_6_ (*p* < 0.05). There were no statistically significant differences in the scores at any of the time points between group N + D and group D (Table 4).

**TABLE 4:** Immediate extubation agitation score and Ramsay score at different time points in each group ( $\bar{x}$ *±s*).

Group	T_3_ (agitation score at tube removal)	T_4_ (RS score)	T_5_ (RS score)	T_6_ (RS score)
C	1.6 ± 0.9	3.5 ± 1.1	1.6 ± 1.0	1.5 ± 1.1
D	0.1 ± 0.1^aa^	4.3 ± 1.0^a^	3.4 ± 1.1^a^	2.4 ± 0.7^a^
N + D	0.0 ± 0.0^aa^	4.2 ± 0.8^a^	3.2 ± 0.9^a^	2.5 ± 0.9^a^

^a^p < 0.05 compared with group C.

^aa^p < 0.01 compared with group C.

### Cough Score at Tube Removal

Compared with group C, the cough score was greatly decreased in group D and group N + D (*p* < 0.01). There were no statistically significant differences in the scores at any of the time points between group N + D and group D (Table 5).

**TABLE 5:** Comparison of cough grading at tube removal among groups.

Group	n	Level 1	Level 2	Level 3	Level 4	Level 5
C	59	5	17	23	9	5
D^aa^	59	48	8	2	1	0
N + D^aa^	60	49	7	3	1	0

^aa^p < 0.01 compared with group C.

## Postoperative Recovery and Rehabilitation

Compared with group C, in group D and group N + D, the ventilator weaning time was shortened (*p* < 0.05), and the tube removal and awakening times were slightly increased; however, the differences were not statistically significant (*p* > 0.05). The length of postoperative hospital stay was decreased in group N + D compared with the other two groups (*p* < 0.05; Table 6).

**TABLE 6:** Comparison of ventilator weaning, laryngeal mask removal, awakening time, and postoperative hospital stay among groups ( $\bar{x}$ *±s*).

Group	Time of ventilator weaning (min)	Time from ventilator weaning to laryngeal mask removal (min)	Time from ventilator weaning to awakening (min)	Postoperative hospital stay (d)
C	37.9 ± 3.4	7.9 ± 3.8	18.9 ± 5.2	7.3 ± 0.8
D	25.2 ± 5.4^a^	8.3 ± 5.2	19.2 ± 4.0	7.1 ± 0.9
N + D	24.7 ± 4.8^a^	8.5 ± 4.7	19.8 ± 4.0	3.6 ± 0.6 ^a^ ^,^ ^b^

a p < 0.05 compared with group C.

b p < 0.05 compared with group C.

## Discussion

Approximately 1,500 children with a tracheal foreign body are admitted to our hospital every year. Among them, children with atelectasis in the perioperative period of foreign body removal are susceptible to lung injury due to hypoxia and reoxidation reaction after pulmonary re-expansion, a stress reaction in the perioperative anesthesia period, and the release of numerous oxygen free radicals and inflammatory factors.

In recent years, the application of dexmedetomidine, an α_2_ adrenergic receptor agonist, for organ protection has been widely used. Dexmedetomidine can activate the α_2_ receptors of the central postsynaptic membrane, reduce sympathetic tone, and increase vagal excitability. It can activate the presynaptic α_2_ receptors in peripheral sympathetic endings, inhibit the release of norepinephrine, and decrease the concentration of plasma catecholamine (Giovannitti et al., 2015). Furthermore, it can stabilize hemodynamics, relieve oxidative stress caused by surgical trauma, (He et al., 2021), reduce the synthesis and release of pro-inflammatory factors (e.g., TNF-α and IL-6) (Xu et al., 2019), and protect the lung through multiple ways, including anti-sympathia and inhibition of apoptosis, oxidative stress, and inflammatory response (Li et al., 2016).

Dexmedetomidine’s effects include analgesia, sedation, and salivation; however, it barely inhibits breathing, (Liu et al., 2021), making it an ideal sedative, and has more obvious advantages for the removal of a foreign body in the bronchus.

According to the literature (Li et al., 2020) and the preliminary experiment (Wang and Jia, 2015), 0.5 μg kg^−1^•h^−1^ was pumped intravenously in the present study; the total dose did not exceed 1 μg kg^−1^, thus remaining within the safety range.

The Cmax could be reached during awakening; this can be effective against agitation during awakening and the cough response during tube removal.

The results of the present study are as follows: there were five cases of hypoxemia in the conventional anesthesia group during awakening; meanwhile, there was no such case in the experimental group. The cycle was more stable in the perioperative period. Bradycardia occurred in only two cases in each group and was quickly corrected.

The following result is consistent with relevant research: intravenous pumping of dexmedetomidine could increase hemodynamics stability in the perioperative anesthesia period and improve postoperative recovery quality (Andersen et al., 2017; Ding et al., 2017). Most adverse reactions of hypotension and bradycardia were transient and easy to correct (Vorobeichik et al., 2017).

The main experimental indicator of the present study was the comparison of TLR-2, TLR-4, TNF-α, and IL-6 before and after the foreign body removal in children with atelectasis; this was conducted to explain the inflammatory mechanism of the non-infected lung injury. Toll-like receptors are “portals” that initiate the inflammatory response, and TLR-2 and TLR-4 are important members of the TLR family; (Bahia et al., 2019); they play an important role in non-infectious lung injury and can activate the intracellular signal pathway and the nuclear factor NF-κB.

As an oxidative stress-sensitive transcription factor, NF-κB is at the core of inflammatory response regulation. After activation, NF-κB can centrally regulate cytokines and other pro-inflammatory mediators (Wang et al., 2021); regulate the gene expression of a series of inflammatory cytokines; participate in the occurrence and development of pulmonary inflammation; and induce TNF-α, IL-1, IL-_6_, IL-_8_, and secondary inflammatory factors to form a “cytokine cascade reaction,” thus initiating lung inflammation.

Compared with the conventional anesthesia group, the two experimental groups for which dexmedetomidine was added had a higher oxygenation index, shorter postoperative ventilator weaning time, and fewer inflammatory factors. These results fully prove the protective effect of dexmedetomidine.

The B-line, also called the “comet tail sign,” is a number of strong parallel echo lines that are gradually adducted and weakened from the lung wall interface to the edge of the screen under pulmonary ultrasound (Ciumanghel et al., 2017). A relative or significant increase in extravascular lung water will lead to an increase in the B-line (Picano and Pellikka, 2016).

In the present study, groups with dexmedetomidine had a lower ultrasound B-line sum than groups without dexmedetomidine, indicating a less extravascular pulmonary fluid content. Due to the influence of the fluid balance on the B-line, the sufficiency of the evidence of dexmedetomidine decreasing the B-line sum depends on the results of the fluid balance monitoring, especially the result of the fluid intake. However, data on fluid balance were not collected in the present study. As there is currently no relevant research on the effect of dexmedetomidine on fluid balance, the fluid balance will be monitored during the future study to prove the effect.

Nalbuphine hydrochloride is a lipophilic semi-synthetic opioid that can bond to κ, δ, and μ receptors in order to show strong κ receptor excitation and μ receptor antagonism; it is comparable to morphine in terms of analgesic effect (Ramstead et al., 2016). Nalbuphine becomes effective 2–3 min after intravenous administration; it has a plasma half-life of 5 h and effectiveness of 3–6 h. It can be used before surgery for anesthesia with a long duration and few adverse reactions (Gonçalves de Freitas et al., 2016; Xu et al., 2019).

Studies show that the preoperative use of nalbuphine can inhibit the patient inflammatory response as well as effectively inhibit surgical trauma (Jiang, 2018). A dose of 0.2 mg kg^−1^ nalbuphine in children without respiratory depression (Wang, 2017) in combination with μ receptors can partially reverse or block opiate-induced respiratory depression.

In this study, the use of 0.2 mg kg^−1^ nalbuphine before operation enhanced the effect and delayed the time of the inflammatory response without prolonging the time of ventilator weaning and awakening. As a result, the inflammatory response factor was low and the oxygenation index was high at 2–24 h after the operation. In addition, the extravascular pulmonary fluid content was reduced and the length of hospital stay was shortened. This may be due to the fact that early use can more effectively prevent central sensitization caused by anesthetic stimulation and surgical trauma as well as inhibit the synthesis and release of TNF-α and IL-6 than those in late use (Gonçalves de Freitas et al., 2016).

It has been reported that retaining spontaneous respiration is mostly used for removing a foreign body from a bronchus. In such cases, muscle relaxants can be used to control breathing.

In the present study, total intravenous anesthesia and the muscle relaxant cisatracurium were used to ensure anesthesia depth and complete muscle relaxation for foreign body removal. Ventilation was used intermittently (ventilation was interrupted only transitorily during foreign body removal) to meet the oxygen supply and reduce adverse reactions caused by anesthesia. There was no breath-holding or airway spasm during the operations. These results are consistent with the relevant literature (Cai et al., 2017).

Remifentanil, a short-acting opioid analgesic, shows stable hemodynamics and no accumulation; this is conducive to rapid postoperative recovery. In the recovery period, the SIMV + PSV mode of the ventilator is gradually weaned to ensure oxygenation as well as reduce cough, man–machine counteraction, and respiratory muscle acting (Cao and Chen, 2018).

The present study has several limitations: there were deficiencies in the sample size, observation indicators, and groupings. In the future, more samples and observation indicators, as well as accurate grouping, will be taken into consideration for an in-depth comparative study.

In conclusion, in children with atelectasis, nalbuphine combined with dexmedetomidine may reduce the release of inflammatory factors during the removal of a foreign body from a bronchus in the perioperative anesthesia period, inhibit oxidative stress reaction, improve the oxygenation index, and protect the lung; it may also lower agitation during awakening and the cough reaction during tube removal. In addition, the circulatory function is stable, and the awakening time is not prolonged, thus accelerating postoperative recovery.

## Data Availability

The original contributions presented in the study are included in the article/Supplementary Material, further inquiries can be directed to the corresponding author.
